# Preparation of High-Strength and High-Rigidity Carbon Layer on Si/C Material Surface Using Solid–Liquid Coating Method

**DOI:** 10.3390/nano15171300

**Published:** 2025-08-22

**Authors:** Xiaoguang Zhang, Wei Wang, Juan Zhang

**Affiliations:** 1 School of Aerospace Engineering, North University of China, Taiyuan 030051, China; 2 College of Mechanical and Electrical Engineering, Nanjing University of Aeronautics and Astronautics, Nanjing 210016, China; 3 Jiangsu E-Ontech Company, Nanjing 211106, China

**Keywords:** Si/C, mechanical property, Young’s modulus, high strength, high rigidity

## Abstract

The application of silicon–carbon (Si/C) composite materials in lithium-ion batteries faces problems regarding volume expansion and surface defects. Although coating is a popular modification scheme in the market, the influence of carbon layer quality on the electrochemical performance of Si/C still needs to be studied. By comparing the carbon layers produced by solid-phase and liquid-phase coating methods, an innovative solid–liquid coating technology was proposed to prepare high-strength and high-stiffness carbon layers, and the effects of different coating processes on the physical, mechanical, and electrochemical properties of the materials were systematically studied. Through physical properties and electrochemical testing, it was found that the solid–liquid coating method (Si/C@Pitch+RGFQ) can form a carbon layer with the least defects and the highest density. Compared with solid-phase coating and liquid-phase coating, its specific surface area (SSA) and carbon increment are the lowest, and the surface carbon content and oxygen content are significantly reduced after solid–liquid coating. Mechanical performance tests show that the Young’s modulus of the carbon layer prepared by this method reaches 30.3 GPa, demonstrating excellent structural strength and elastic deformation ability. The first coulombic efficiency (ICE) of Si/C@Pitch+RGFQ reached 88.17%, the interface impedance (23.2 Ω) was the lowest, and the lithium-ion diffusion coefficient was significantly improved. At a rate of 0.1 C to 2 C, the capacity retention rate is excellent. After one hundred and a half-cell cycles, the remaining capacity is 1420.5 mAh/g, and the capacity retention rate reaches 92.4%. The full-cell test further proves that the material has a capacity retention rate of 82.3% and 81.3% after 1000 cycles at room temperature and high temperature (45 °C), respectively. At the same time, it has good rate performance and high-/low-temperature performance, demonstrating good commercial application potential. The research results provide a key basis for the optimized preparation of the surface carbon layer of Si/C composite materials and promote the practical application of high-performance silicon-based negative electrode materials.

## 1. Introduction

In today’s era of booming technology, the innovation of energy storage technology has become the core driving force for progress in many fields [1,2,3]. Lithium-ion batteries are one of the most widely used energy storage devices in modern society. They have significant advantages. These include large capacity, a wide operating temperature range, a long cycle life, no memory effect, and environmental friendliness. They have been deeply integrated into multiple key fields such as portable electronic devices, transportation vehicles, energy storage power stations, and aerospace [4,5]. In lithium-ion batteries, the performance of negative electrode materials is crucial to the overall battery performance. It directly relates to key indicators like energy density, cycle life, and safety performance [6,7,8,9].

At present, among various lithium-ion battery negative electrode materials, the graphite negative electrode occupies the main market with its relatively mature technology and stable performance [10,11,12]. However, with the increasing demand for energy density in various energy storage scenarios, the limitations of graphite anodes in energy storage are becoming increasingly prominent [13]. The theoretical capacity of graphite in lithium batteries is only 372 mAh/g, which no longer meets the growing demand for high-capacity and high-energy-density batteries. So, researchers have focused their attention on silicon-based materials. Silicon materials have a theoretical specific capacity of up to 4200 mAh/g. They are regarded as the most promising new-generation negative electrode materials for lithium-ion batteries [14,15]. This brings new hope for breaking through the performance bottleneck of existing batteries. However, in the actual application process, silicon materials face many severe challenges. Despite their advantages in various performance indicators, two key issues restrict their use as lithium-ion battery negative electrode materials: a volume expansion of ~300–400% during lithiation and poor electrical conductivity [16,17,18,19]. Therefore, how to optimize silicon negative electrode materials has become one of the research hotspots. In order to reduce the volume expansion of silicon, researchers attempted to mix silicon nanomaterials with different carbon materials (graphite, carbon nanotubes, graphene, pyrolytic carbon, etc.) to form silicon–carbon (Si/C) composite materials [16,20,21,22,23]. Silicon active materials can provide high capacity and good energy density, while carbon conductive materials can provide good conductivity and thermal stability. The carbon matrix of Si/C composite materials not only improves the conductivity of the material but also provides some space for the expansion of silicon. Therefore, Si/C composite anode materials exhibit good comprehensive performance. They can effectively improve the cycle life of batteries. As a result, they have become a new type of anode material for lithium-ion batteries. However, despite demonstrating excellent performance, the widespread application of Si/C composite materials in commercial lithium-ion batteries still has limitations [24,25]. Most designed high-performance Si/C composite materials have numerous surface defects. These defects make it easy for electrolytes to penetrate and react with internal silicon, while surface silicon particles are prone to directly react with electrolytes. The result is volume expansion of ~200% and significant volume changes during cycling. The solid electrolyte interface (SEI) film is unstable, leading to low initial coulombic efficiency, and further optimization of the material surface is needed [26].

To address the volume expansion of Si/C composite materials, two main methods have been used. These methods aim to improve the structural stability and electrochemical performance of silicon anodes. They are widely recognized as the most studied and effective approaches in this field. The first method is to modify silicon materials into different nanoscale structures, such as nanoparticles, nanowires, nanotubes, nanofilms, and nanoporous materials [27,28,29,30]. The purpose is to minimize their volume effect and the possibility of crushing. Another method is to coat the surface of Si/C composite material with a dense layer of carbon [31,32]. The carbon layer has high conductivity, which can solve the problem of low conductivity in Si/C composite materials. It can also encapsulate Si/C composite materials inside the shell. The carbon layer exhibits a certain stiffness. It forms an SEI film on its surface, which suppresses electrolyte erosion and damage to Si/C composite materials. This suppression enhances the cycle life, and under conditions that depend on the carbon layer structure and preparation process, the rate performance of the material can be improved by 20–50%. Due to the high cost and long experimental period of modifying silicon materials, carbon coating is one of the effective methods for solving the expansion of silicon negative electrodes [33,34]. Studies have shown that as long as the outer layer of an Si/C composite material is carbon coated, it can act as a buffer layer, thereby further reducing the volume expansion of silicon–carbon negative electrodes. However, regardless of the method of carbon coating, Si/C composite materials need to have good surface quality, including a small specific surface area to reduce surface activity. In order to prevent direct contact between the exposed silicon on the surface of Si/C composite materials and the electrolyte, a uniform and dense carbon layer is introduced to improve surface strength and limit the overall expansion of composite particles. This design is particularly critical in commercial lithium-ion batteries (LIBs), where the limited electrolyte amount exacerbates the risk of excessive consumption caused by uncontrolled reactions between exposed silicon and electrolyte. Active materials with poor surface quality have higher surface activity and often have stronger reactivity with electrolytes. At the same time, the electrolyte enters the interior of Si/C composite material through the pores on the surface and is absorbed by the composite material, resulting in a reduction in effective external electrolyte. The rapid consumption of electrolytes due to reactions and absorption can seriously weaken the life cycle of batteries. This problem seriously restricts the development of silicon-based negative electrode materials and is a key technological bottleneck that needs to be overcome. Many studies have mentioned the importance of coating carbon layers, but few have delved into the impact of the density and integrity of carbon layers on material properties. In the field of coating methods for Si/C composite negative electrode materials, early research on double coating layers was mostly limited to the simple superposition of two coating processes [5] and failed to deeply integrate and synergistically regulate the intrinsic characteristics of carbon sources. In addition, most existing studies rely on multi carbon source chemical vapor deposition technology to construct carbon coating layers [35,36,37]. This method not only has the problem of high preparation cost, but the material synthesis process is also influenced by multiple factors such as reaction atmosphere, precursor concentration, temperature gradient, etc., which makes it difficult to accurately control the consistency of the product. At the same time, CVD processes generally face industrialization bottlenecks such as low production capacity and poor safety of high-temperature reaction systems. In other dual carbon coating systems, some studies have used carbon nanotubes as auxiliary carbon sources [38]; although they can optimize conductivity, they significantly increase the preparation cost of the material and limit its prospects for large-scale market applications. In the study of preparing surface carbon layer using the solid–liquid coating method, some schemes use carbon sources with toxicity risks [39], which may pose potential health hazards to operators. Another study requires complex solvent-mediated reactions to achieve encapsulation [40], resulting in lengthy preparation processes and increased process complexity, which is not conducive to industrial scaling up. Therefore, a new coating technology solution that can achieve deep synergy of carbon source characteristics, strong process controllability, low preparation cost, safe operation, and adaptability to large-scale production is needed to address the pain points of existing coating technologies.

Therefore, according to the needs of practical applications, this article uses three methods: solid-phase coating, liquid-phase coating, and solid–liquid coating to prepare the surface carbon layer of Si/C composite materials. After testing to obtain the Young’s modulus of the material, it was verified which of these three coating methods can form a carbon layer with higher compressive strength. Finally, the long-term cycling stability of the material was tested using a cylindrical 18650 full cell combined with a ternary cathode material, LiNi_0_._8_Co_0_._1_Mn_0_._1_O_2_. This provides an effective standard for preparing carbon layers on the surface of Si/C composite materials, promoting the commercialization of Si/C composite lithium-ion batteries.

## 2. Experiment

The Si/C material used in this article was prepared by our research group. It uses graphite nanosheets as the framework carrier to uniformly disperse nano silicon particles and carbon nanotube materials. Graphite nanosheets, carbon nanotubes, and nano silicon (SiNPs, D_50_ = 87 nm) were provided by Jiangsu E-ontech, Nanjing, China. The high specific surface area of graphite nanosheets and carbon nanotubes provides sufficient space for the attachment of nano silicon particles.

### 2.1. Material Preparation

**Figure 1:**
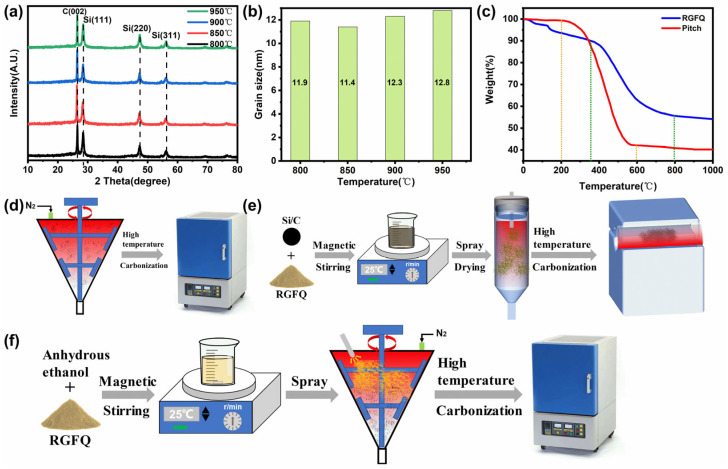
Different temperatures: (**a**) XRD patterns and (**b**) average grain size. (**c**) Thermogravimetric curve of RGFQ and coal tar pitch powders, (**d**) dynamic solid-phase coating process, (**e**) dynamic liquid-phase coating process, and (**f**) dynamic solid–liquid-phase coating process.

Due to the presence of silicon particles in the material, which are covalently bonded crystals, the atomic diffusion ability is enhanced at high temperatures. The elastic strain energy stored after deformation drives grain boundary migration, resulting in grain growth. When the temperature exceeds the recrystallization temperature, silicon grains gradually transform from small, deformed structures to coarse equiaxed crystals, and the grain boundary density decreases. Carbon undergoes grain boundary defects at high temperatures, with grains growing in the basal plane (0001) direction and a slight shrinkage in interlayer spacing. As the difference in thermal expansion coefficients between Si and carbon (Si is about 2.6 × 10^−6^/°C, graphite is about 1 × 10^−6^/°C) generates interface thermal stress at high temperatures, promoting the preferential nucleation of silicon near the interface and forming interface-induced recrystallization, the grain size of the material increases [41]. Therefore, it is necessary to obtain appropriate carbonization temperature values to maintain the optimal grain size of the material during the high-temperature reaction process and minimize the overall impact on the material. In order to determine the carbonization temperature of Si/C composite materials and control the grain size of the material, the Si/C composite materials are first placed in a furnace and heated to 800, 850, 900, and 950 °C, respectively, under the protection of high-purity nitrogen gas. After 2 h of insulation, the materials are taken out at room temperature. Figure 1a shows the XRD patterns of Si/C composite materials at four different temperatures. It can be seen from the figure that the diffraction peaks of 2 Theta for Si in Si/C composite materials at 28.44°, 47.31°, and 56.12° correspond to the crystal planes of Si (111), Si (220), and Si (311), respectively, at different temperatures. The diffraction peak of the graphite C (002) crystal plane appears at 26.54°. According to the XRD pattern in Figure 1a, the average grain size of Si/C composite material at four temperatures in Figure 1b was calculated using the Scherrer formula. Figure 1b visually presents the relationship between grain size and temperature. It can be seen from the figure that at 850 °C, the average grain size of Si/C composite material is the smallest, at 11.4 nm. Compared to other temperature conditions, the grain size at this temperature is better (smaller), thus minimizing the volume change during charge–discharge cycles. The coating temperature of the Si/C composite material is selected as 850 °C.

The dynamic solid-phase coating process is shown in Figure 1d. The associated process is as follows: Weigh 1 kg of Si/C composite material and 0.25 kg of pitch according to the mass ratio of 8:2, and put them into the V-shaped Covering (VC) fusion machine. The main shaft disperses and mixes the pitch and Si/C composite material at a speed of 650 r·min^−1^ for 2.5 h. Then introduce high-purity nitrogen gas (purity 99.999%) into the VC melting machine at a rate of 300 mL/min for oxygen purging. When the oxygen content is less than 100 ppm, the first stage is started, with heating up to 300 °C at a heating rate of 8 °C·min^−1^ for 3 h, and the main shaft speed is reduced to 350 r·min^−1^ for stirring. You can see the pitch thermogravimetric curve in Figure 1c, at which point the temperature of the pitch begins to soften and has a certain degree of fluidity. Due to the complete carbonization of pitch at 600 °C and complete carbonization at 700 °C, the remaining carbon is completely carbonized by 42%. So, the second stage was started with a heating rate of 3 °C·min^−1^ to raise the temperature to 700 °C and maintain it for 1.5 h. The spindle speed was reduced to 2000 r·min^−1^ for low-speed stirring. Finally, the third stage spindle speed was reduced to 50 r·min^−1^, and the temperature was lowered to 20 °C at a cooling rate of 8 °C·min^−1^ for 1 h to obtain carbon-coated particles. The material was then placed in a box furnace and heated to 850 °C at a rate of 5 °C·min^−1^ at a rate of 200 mL/min under high-purity nitrogen protection for 2 h for carbonization. After cooling it down to room temperature, the prepared material was taken out. At this point, a stable carbon layer had formed on the surface of the material, marked as Si/C@Pitch.

The specific process of the dynamic liquid-phase coating method is shown in Figure 1e, where Si/C: ReGuFenQuan (RGFQ) = 92:8. To conduct the experiment, first weigh 250 g of anhydrous ethanol and pour it into a beaker. Then weigh 27.8 g of RGFQ and place it in the beaker. Place the beaker in a magnetic stirrer and stir at a speed of 1200 r·min^−1^ for 1 h to disperse the RGFQ, obtaining a 10 concentration RGFQ solution. Afterwards, 250 g of anhydrous ethanol was weighed and poured into a beaker. A total of 320 g of Si/C negative electrode material was placed in a stirred beaker. The Si/C composite material was dispersed by stirring with a magnetic stirrer at a speed of 1500 r·min^−1^ for 2 h. Finally, the dispersed RGFQ slurry and Si/C negative electrode material slurry were poured into the beaker and stirred with a magnetic stirrer at a speed of 1800 r·min^−1^ for 2.5 h to obtain a mixed slurry. The slurry was placed at the feeding port of HF-6000Y spray dryer, stirred while the peristaltic pump entered the spray gun at 30 r·s^−1^. Under the protection of high-purity nitrogen at a speed of 300 mL/min, the spray gun sprayed the slurry into the spray drying tower at a pressure of 0.8 Mpa. The temperature of the spray drying tower was set to 180 °C. The atomized material was dried by contacting hot air, so that the solvent in the material was evaporated and dried into small powder particles. Then the gas generated by the air compressor of the equipment would send the small powder particles through the cyclone separator and fall into the collection bottle. The thermogravimetric image of RGFQ in Figure 1c shows that starting from room temperature, the mass of RGFQ gradually decreases with the continuous increase in temperature. When the temperature reaches around 150 °C, RGFQ begins to react, which involves complex group condensation reactions and results in weight loss of RGFQ at this stage. When the temperature is within the range of 410 °C to 600 °C, the weight loss is significant, which represents the high-temperature pyrolysis carbonization process of RGFQ. During its pyrolysis carbonization process, water and a small amount of carbon dioxide are first produced. Then, as the temperature continues to rise, it decomposes to produce methane, benzene, and its derivatives, and finally produces hydrogen and carbon monoxide. These changes cause significant mass loss in RGFQ. At around 850 °C, the weight loss of RGFQ stops, indicating that the pyrolysis carbonization reaction has basically ended. Therefore, a moderate heat treatment temperature of 850 °C can completely carbonize RGFQ. So, the collected materials were placed in a tube furnace for carbonization at 850 °C, and the RGFQ was decomposed to obtain a layer of carbon coating on the surface of Si/C composite material, marked as Si/C@RGFQ.

The dynamic solid–liquid coating process is shown in Figure 1f. The specific process is as follows: according to Si/C:Pitch:RGFQ = 94.2:5:0.8, weigh 29.7 g of anhydrous ethanol and pour it into a beaker. Then weigh 16 g of RGFQ and place it in the beaker. Place the beaker in a cantilever electric stirrer and stir at a speed of 200 r·min^−1^ for 0.5 h to disperse the RGFQ, obtaining a 34.8% concentration RGFQ solution. After the RGFQ is completely dissolved in anhydrous ethanol, stir at a speed of 1800 r·min^−1^ for 1 h to obtain a solution. Weigh 1884 g of Si/C composites and 100.2 g of pitch, respectively, and put them into the VC fusion machine. Under the protection of high-purity nitrogen gas (99.999% purity) at a speed of 300 mL/min, the main axis is first rotated at a speed of 600 r·min^−1^ to disperse asphalt and Si/C composite materials and mix for 2 h. Then, open the first section to heat the mixture up to 250 °C at the heating rate of 3 °C·min^−1^ for 1.5 h, while the main shaft speed is reduced to 400 r·min^−1^ for mixing. Open the second section to heat the mixture up to 700 °C at the heating rate of 3 °C·min^−1^, while the main shaft speed is reduced to 150 r·min^−1^ for low-speed mixing. Then, open the peristaltic pump of the spray dryer RGFQ solution at 20 r·s^−1^ introduce the spray gun. Under the protection of high-purity nitrogen at a speed of 200 mL/min, the spray gun splashes the slurry from the nozzle onto the material at a pressure of 0.8 Mpa. When the solution is sprayed, keep it warm for 2 h. Finally, turn on the third section of the main shaft and reduce the speed to 30 r·min^−1^ at 5 °C·min^−1^. The cooling rate is reduced to 20 °C and kept for 1 h. Then, the material is placed in a box furnace and carbonized at 850 °C to obtain carbon-coated particles, labeled as Si/C@Pitch+RGFQ.

### 2.2. Physical and Chemical Characterizations

The particle size distribution of the micron-scale materials was measured using a Laser Particle Size Analyzer (LPSA) (Omec LS-609, OMEC, Zhuhai, China), providing data such as D10, D50, and D90. The external morphology and internal structure of the samples were examined using scanning electron microscopy (SEM) (Hitachi S-4800, HITACHI, Kyoto, Japan), X-ray diffraction (XRD) (D/max2500PC, Rigaku Corporation, Osaka, Japan), and Raman spectroscopy (Thermo Fisher DXRxi, Thermo Fisher Scientific Inc., Osaka, Japan). X-ray photoelectron spectroscopy (XPS) (AXIS Supra, Kratos Analytical Ltd., Kyoto-fu, Japan) was employed to analyze the elemental composition and chemical states of the surface elements. The carbon content of the samples was determined using a high-frequency infrared carbon–sulfur (C-S) analyzer (N-HW2000A, Nanjing Ningbo Analytical Instrument, Nanjing, China). The specific surface area (SSA) of the samples was measured by a multipoint BET nitrogen adsorption–static capacity method (JWGB BK112, JWGB, Beijing, China). Electrical conductivity was tested using a polarizer resistance tester (BER1300, IEST, Xiamen, China), and interface impedance was measured using an electrochemical workstation (CHI660E, Shanghai Chenhua Instrument Co., Ltd., Shanghai, China). AFM (SPM9700, Shimadzu, Kyoto, Japan) is used to test the force-displacement curve of the particles. Force curve tests were conducted on three materials using NSG-10 single-crystal silicon probes with a tip curvature radius of approximately 10 nm and an elastic coefficient (Kc) range of 5.1–50.9 N/m. The software Nano Scope Analysis software for v1.5.0 was used to analyze the fitted force curve, and the Young’s modulus of the sample was calculated according to the elastic mechanical contact model. The compression performance of the prepared material was tested using a PRCD3100 (IEST, Xiamen, China) compacted powder resistance meter. The electrochemical performance of the CR2032-type coin half cells and 18650-type cylindrical full cells was evaluated using the Land Battery Test System (CT2001A, LANHE, Wuhan, China).

### 2.3. Electrochemical Characterizations

CR2032-type coin cells were used for half-cell testing, with the following production process: Active materials (samples), carbon black conductive agent (SP), sodium carboxymethyl cellulose (CMC), and polymerized styrene-butadiene rubber (SBR) were mixed in a mass ratio of 95.8:1:1.4:5.6. The resulting slurry was evenly coated onto a 15 μm thick copper foil. After natural drying at room temperature for 8 h, the coated copper foil was transferred to a vacuum oven at 80 °C and dried for 10 h. The foil was then compacted to 75% of its original thickness using a roller press, punched, and cut into 13 mm diameter disks. The half cells were assembled in a high-purity argon glove box, using lithium metal foils as the counter electrode and polypropylene macroporous films (Celgard 2400, Celgard LLC, Charlotte, NC, USA) as the separator. The electrolyte was a 1 M LiPF_6_ solution in a mixture of ethylene carbonate (EC), dimethyl carbonate (DMC), and ethyl methyl carbonate (EMC) (1:1:1 by volume) with 5 wt% fluorinated ethylene carbonate (FEC).

For the 18650-type cylindrical batteries used in full-cell testing, the production process was as follows: Active materials (samples), SP, CMC, SBR, and deionized water were mixed in a mass ratio of 100:1:1.4:1.4:100 to form the anode slurry. The cathode active material used was LiNi_0_._8_Co_0_._1_Mn_0_._1_O_2_ (NCM811), with the specific formulation being SP: polyvinylidene fluoride (PVDF): NMP: NCM811 = 1.5:1.5:37:100. The cathode slurry was uniformly coated on both sides of a 15 μm thick aluminum foil, while the anode slurry was coated on an 8 μm thick copper foil. The areal densities of the cathode and anode electrodes were 400 and 200 g·m^−2^, respectively, with compaction densities of 2.87 and 1.65 g·cm^−3^. After slitting, welding lugs, coiling, shelling, welding, rolling grooves, and drying (at 80 °C under vacuum for 12 h), the batteries were filled with electrolyte and underwent further assembly processes to complete the final cylindrical batteries.

## 3. Result and Discussion

### 3.1. Physical Performance Analysis

**Figure 2:**
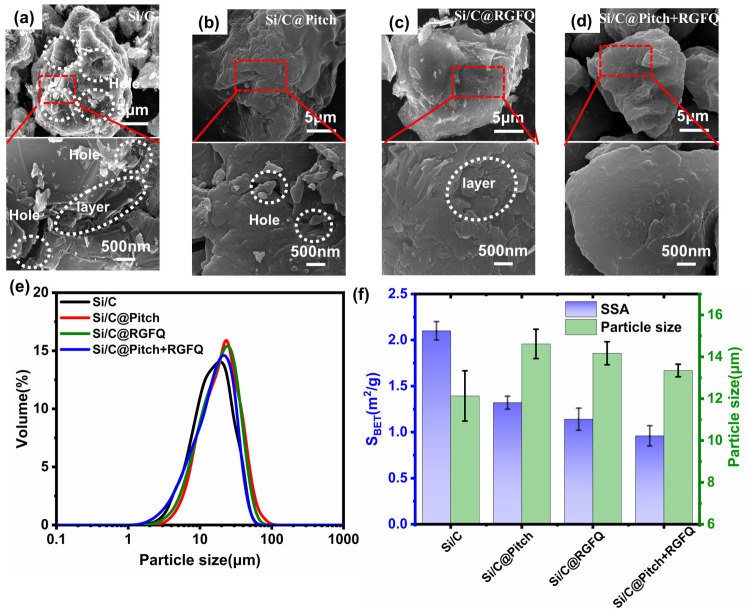
SEM surface morphology of (**a**) Si/C, (**b**) Si/C@Pitch, (**c**) Si/C@RGFQ, and (**d**) Si/C@Pitch+RGFQ. (**e**) Particle size distribution curves, (**f**) SSA (left vertical axis), and median particle size (D_50_) (right vertical axis) curves.

Figure 2a–d show the surface morphology of all samples at different magnifications. Figure 2a shows the rough surface of the original Si/C particles, indicating the presence of many small particles, cracks, pores, and layered defects. After coating with different carbon sources, the number of small particles and pores in Si/C@Pitch (Figure 2b), Si/C@RGFQ (Figure 2c), and Si/C@Pitch+RGFQ (Figure 2d) was significantly reduced, resulting in a smoother surface. This indicates that the coating treatment effectively modifies surface defects. In Figure 2c, it can be seen that there are still many step-like and layered defects on the surface of Si/C@RGFQ, as well as some blocky particles. In addition, Figure 2d shows that there are almost no visible pores and cracks on the surface of Si/C@Pitch+RGFQ, indicating that the layered defects have been sufficiently filled with carbon to make the particle surface smoother. The prepared material was subjected to particle size testing, and the particle size curve distribution in Figure 2e was obtained. It can be seen from the figure that the volume proportion of Si/C composite material after coating is higher than that before coating, and the particle size distribution after coating is narrower, indicating that the uniformity and consistency of the coated material are higher than those before coating, further optimizing the overall performance of the material [42]. The coating treatment significantly changed the surface defects, but the surface quality prepared with different process parameters showed significant changes, which require further analysis. Si/C composite materials require a uniform carbon layer with low SSA and the lowest carbon content, as excessive carbon can reduce the specific capacity of the material. Figure 2f shows the relationship between the specific surface area (left vertical axis) and the median particle size D_50_ (right vertical axis) of the sample, which proves that Si/C@Pitch has a larger median particle size D_50_ when the specific surface area decreases at a lower rate, indicating a negative correlation between the median particle size D_50_ and the rate of decrease in the specific surface area of the sample. At the same time, carbon coatings have the ability to adhere Si/C particles at the nanometer level to the surface of micrometer-level particles, thereby reducing the SSA, reducing particle distribution, and increasing the median particle size (D_50_) of the sample. The median particle size of the solid-phase-coated sample increased by about 2 μm, which is related to larger pitch particles. Pitch particles can bind with larger particles and even clump together. This indicates that the carbon layer formed by pitch has the lowest quality, with many uncovered pores and discontinuous carbon layers. The median particle size of the solid–liquid coated sample increased by about 1 μm, indicating that some agglomeration phenomena still occurred.

### 3.2. Morphological Analysis

**Figure 3:**
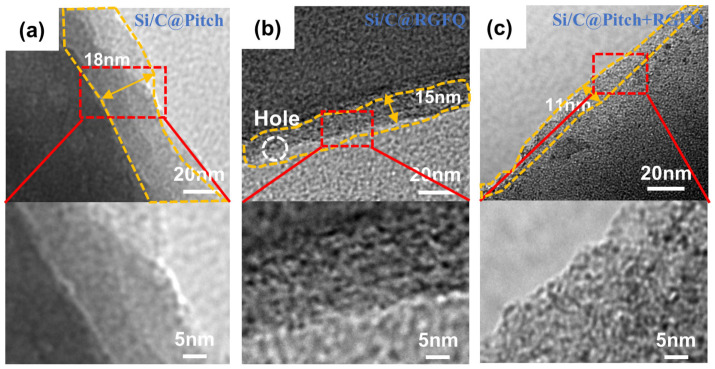
TEM images of (**a**) Si/C@Pitch, (**b**) Si/C@RGFQ, and (**c**) Si/C@Pitch+RGFQ at different magnifications.

Figure 3a–c show the internal structure of the carbon layer on the surface of Si/C@Pitch, Si/C@RGFQ, and Si/C@Pitch+RGFQ materials, with the same magnification for each sample. The figure shows that the carbon layer of Si/C@Pitch is relatively thick, at 18 nm, which is also related to the influence of solid-phase coating. The carbon layer thickness of Si/C@Pitch+RGFQ is only 11 nm. The enlarged image in the figure shows that the carbon layers of the prepared carbon layers are all amorphous.

**Figure 4:**
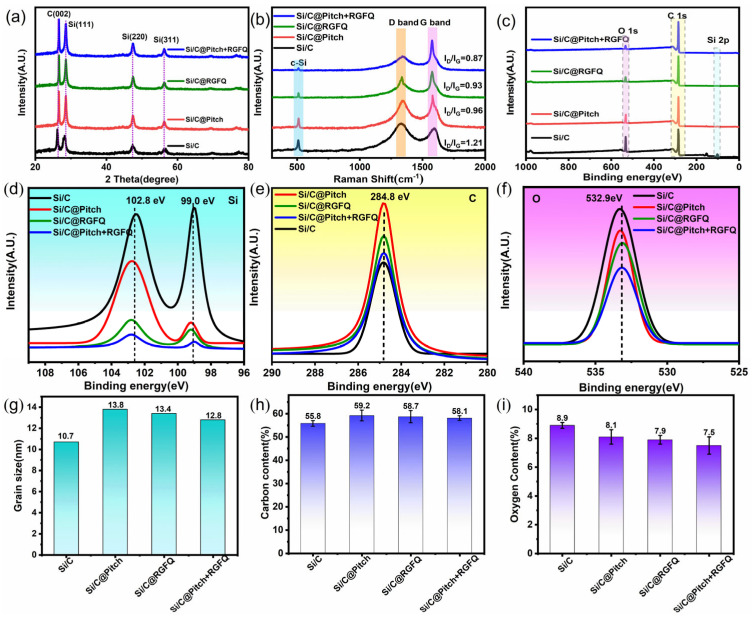
(**a**) XRD spectrum, (**b**) Raman spectrum, (**c**) XPS spectrum, (**d**) Si 2p absorption peak, (**e**) C 1s absorption peak, (**f**) O 1s absorption peak of all samples, (**g**) average grain size, (**h**) carbon content, and (**i**) oxygen content.

An easily overlooked issue is that the high efficiency of carbon layer formation on the surface shortens the duration of high temperature, which is crucial for improving the cycling performance of Si/C composites. Due to the movement of lithium ions at different rates on different crystal planes, crystalline silicon undergoes anisotropic expansion, while amorphous silicon exhibits isotropic expansion due to the absence of crystal planes [43,44]. Therefore, during the expansion process of lithium insertion, crystalline silicon is more prone to fracture than amorphous silicon [45]. The silicon nanoparticles of Si/C composite materials in this article have the characteristics of high amorphization and small grain size. However, under high-temperature treatment, the high temperature can lead to recrystallization of the internal amorphous state and gradual growth of grains, resulting in poor ability to suppress expansion and making the material more prone to rupture. The performance of silicon nanoparticles directly affects the performance of Si/C composite materials, leading to a shortened cycle life. Figure 4a shows the XRD patterns of all samples. The diffraction peaks of Si’s 2 Theta at 28.4°, 47.3°, and 56.1° correspond to the (111), (220), and (311) crystal planes, respectively. The diffraction peak of the graphite C (002) crystal plane appears at 26.5°. It can be observed that after high-temperature treatment using solid-phase coating, solid–liquid coating, and solid–liquid coating processes, the characteristic peak intensity of Si increases and the peak width slightly decreases. However, in the solid–liquid coating process, the characteristic peak intensity of Si is higher and the peak width is smaller, indicating that the internal amorphous Si has weak recrystallization behavior under high temperature. Therefore, according to the analysis conclusion, adopting the solid–liquid coating process can effectively reduce the impact of high temperature on silicon, which is beneficial for reducing the damage to material properties. There is no peak of silicon carbide (Si/C) in the figure, indicating that Si does not react with carbon at such high temperatures. Figure 4g shows the average grain size of the samples. It can also be seen from the figure that high temperature has a small impact on silicon in the solid–liquid coating process, with an average grain size of 12.8 nm.

The density of the carbon layer formed on the surface directly affects other surface properties. For example, a higher density of carbon layer means fewer defects, a smaller specific surface area, and a stronger carbon layer, which can better suppress particle expansion. At the same time, the thickness of the carbon layer will be more uniform, and the surface silicon content will be lower. Therefore, comparing the degree of defects in the carbon layer is more meaningful than simply comparing the thickness of the carbon layer. Figure 4b shows the Raman spectrum of the sample. The displacements at 1350 and 1580 cm^−1^ are the D and G bands of carbon material, respectively [46]. From the calculation results, it can be seen that the I_D_/I_G_ value of the raw material Si/C is the highest (1.21), and the I_D_/I_G_ values decrease after carbon coating, indicating that carbon defects were repaired to some extent. In addition, the I_D_/I_G_ value of Si/C@Pitch+RGFQ is the lowest (0.87), which once again indicates that the carbon layer formed by solid–liquid coating has the least defects and the highest density [47,48].

Both carbon and silicon materials produce SEI films when in contact with liquid electrolytes, but the volume change of carbon materials is small, resulting in a thin SEI film, while the volume change of silicon materials is greater, and the formed SEI is prone to rupture and rebuild to a higher thickness [49]. Therefore, it is crucial to coat Si/C composite materials with appropriate carbon layers of silicon nanomaterials to prevent direct contact between silicon and electrolytes. Surface silicon content is a key evaluation indicator in industrial applications, and XPS scanning depth is usually within a few nanometers, making it a valuable tool for particle surface element analysis. The XPS spectra of all materials are shown in Figure 4c. From the figure, it can be seen that the sample contains the elements Si, C, and O. In the magnified spectrum of Si 2p in Figure 4d, the peak with a binding energy of 99.0 eV is the Si-Si bond, while the peak with a binding energy of 102.8 eV is mainly attributed to SiOx, indicating that the surface of Si nanoparticles in Si/C has undergone partial oxidation [50]. From these figures, it can be seen that the Si 2p absorption peak of the raw material Si/C is the highest among all samples (8.7%) and significantly decreases after coating, indicating that the bare Si on the surface of the raw material is covered by the deposited carbon layer. The Si particles exposed on the surface of Si/C composite material after carbon coating are covered by the prepared carbon layer. Therefore, the peak intensities of the Si 2p absorption peaks of the prepared materials Si/C@Pitch, Si/C@RGFQ, and Si/C@Pitch+RGFQ have all decreased. Meanwhile, the intensity of the Si 2p peak in Si/C@Pitch is relatively high, indicating that the solid-phase coating method has a poor coating effect and incomplete coverage, resulting in more Si particles remaining on the Si/C surface. Therefore, the valence state of Si/C@Pitch is significantly higher than other samples. At the same time, the I_D_/I_G_ (1.21) of Si/C@Pitch in Figure 4b is higher than that of other samples, which also proves that there are more carbon layer defects formed by pitch coating. The carbon layer with many defects has high chemical activity, so the valence state of Si is high. Meanwhile, the intensity of the Si 2p peak in Si/C@Pitch+RGFQ is the lowest, indicating that the solid–liquid coating method can cover more defects and reduce the residual Si particles on the Si/C surface. The lower the surface silicon content, the better the material performance; however, considering production costs, the surface silicon content is usually required to be less than 5%. In this case, the solid–liquid coating method has a significant advantage in reducing the surface silicon content when comparing coating methods. Figure 4e shows the high-resolution spectrum of material C 1s, where the peak with a binding energy of 284.8 eV is the C-C bond. It can be seen that the intensity of the C 1s absorption peak of the raw material Si/C is the lowest, and the intensity of this peak increases after coating treatment, confirming the gradual increase in surface carbon content. Due to the highest carbon content in Si/C@Pitch, the peak binding energy at 284.8 eV is higher. Figure 4f shows the high-resolution spectrum of material O 1s, where the peak with a binding energy of 532.9 eV is the Si-O bond [51]. This figure indicates that carbon coating is always the most effective way to reduce the surface oxygen content of the material. The specific test results of oxygen content in Figure 4i also show that compared with the other two coating methods, the carbon layer on the surface of the material prepared by solid–liquid coating method is more uniform and complete, which reduces the direct contact between the material and oxygen. The oxygen content is the lowest (8.1%), and excessive oxygen content may cause electrolyte decomposition, reduce the stability of the battery, and lead to adverse electrochemical reactions. A lower oxygen content can avoid the co insertion of lithium ions and solvents, suppress electrolyte decomposition, improve the reversible capacity, cycling stability, and battery rate performance of the material, enhance the first coulombic efficiency of the material, improve the conductivity and cycling stability of the material, and avoid the occurrence of oxidation reactions [52,53]. The carbon content of all samples before and after coating treatment was also tested using a C-S analyzer, and the difference between the two is the carbon increment, which represents the amount of carbon added onto the material surface by the coating process. Based on the XPS results in Figure 4e, it was found that the peak intensity of C 1s increased after coating. Therefore, the accurate carbon content of all samples before and after coating treatment was tested using a C-S analyzer. Figure 4h shows the carbon content results of all samples. From the graph, it can be seen that the carbon content of the Si/C composite material itself is 55.8%, with Si/C@Pitch having the highest carbon content (59.2%). A higher carbon content indicates that the sample is covered with the most carbon.

### 3.3. Mechanical Properties

**Figure 5:**
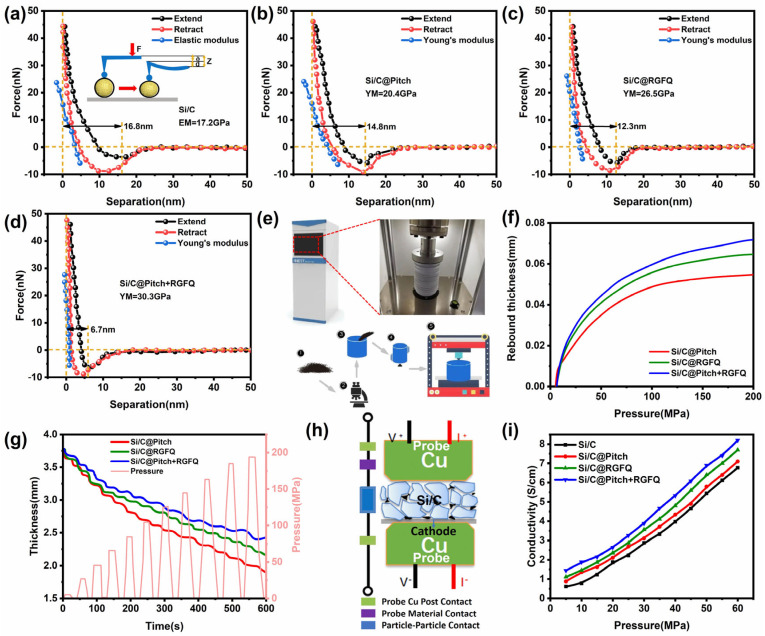
Force curve diagram for (**a**) Si/C, (**b**) Si/C@Pitch, (**c**) Si/C@RGFQ, and (**d**) Si/C@Pitch+RGFQ. (**e**) Schematic diagram of PRCD3100 compaction powder resistance meter and compression performance testing function. (**f**) Curve of rebound thickness difference with pressure variation. (**g**) Curve of material thickness variation with increasing pressure. (**h**) Schematic diagram of BER resistance tester testing. (**i**) Conductivity of materials.

Assuming that Si/C particles are uniform spheres with the same surface and internal properties, the mechanical properties of this uniform material can be analyzed by atomic force microscopy (AFM) [54]. Due to the micro scale size of the coated material, traditional testing instruments and characterization methods have significant limitations in achieving precise manipulation and characterization of individual material particles. In contrast, AFM technology exhibits unique advantages with its sub nanometer level position manipulation accuracy and piconewton level mechanical measurement accuracy [55,56]. In addition, AFM technology can also conduct in-depth research on the mechanical and electrical properties under in situ conditions, which makes it highly valuable for the precise manipulation and characterization of nanoscale materials [57]. The schematic diagram of the particle force curve measured by an AFM probe is shown in Figure 5a. The force–displacement curve of the sample was recorded. We conducted the test at a dew point of −35 °C at room temperature. We tested five different particles for each sample and recorded three force curves for each particle. The results are shown in Figure 5a–d. The extension curve (black line) represents the probe approaching the sample, while the retraction curve (red line) represents the probe leaving the sample. We analyzed the fitted effective force curve data using NanoScope Analysis software and calculated the compressive Young’s modulus of the sample based on the elastic contact model. From the graph, it can be seen that the Young’s modulus values of Si/C, Si/C@Pitch, Si/C@RGFQ, and Si/C@Pitch+RGFQ are 17.2, 20.4, 26.5, and 30.3 GPa, respectively. Among them, the Si/C composite material has the smallest Young’s modulus, and after being coated using different methods, a carbon layer is formed on the surface of the material. The carbon material itself has a higher Young’s modulus. After encapsulation, carbon is introduced as the second phase, which is equivalent to adding a high-modulus “skeleton” to the Si matrix. Through load transfer, the overall deformation resistance of the composite material is enhanced. Therefore, carbon coating can improve Si/C interface bonding and reduce interface defects. On the one hand, a dense carbon layer fills the surface defects of Si particles, making the internal structure of the material more uniform, making the stress transmission smoother, and reducing the “stress concentration local deformation” caused by defects. On the other hand, the interfacial bonding force between carbon and Si is enhanced, and when subjected to external forces, the carbon phase can more effectively constrain the deformation of the Si phase, synergistically increasing the modulus. At the same time, there is a significant difference between the tensile and contraction curves of Si/C@Pitch and Si/C@RGFQ, indicating that they have undergone plastic deformation, with a much greater amount of deformation than Si/C@Pitch+RGFQ. The larger the Young’s modulus, the higher its ability to constrain the expansion of silicon, and the less likely the material is to break. In addition, the stretching curve of Si/C@Pitch+RGFQ is relatively small, and the stretching curves overlap uniformly, indicating that Si/C@Pitch+RGFQ material can undergo elastic deformation under appropriate force, and the structural strength of the prepared sample is higher than that of other samples. Only materials with good elastic deformation can recover their original shape after lithiation expansion and delithiation contraction. The results show that the solid–liquid coating method can obtain a dense carbon layer, which can improve the structural strength of the material. The Young’s modulus of the obtained carbon layer is higher than that of the solid- and liquid-phase-coated carbon layers. Si/C@Pitch+RGFQ can greatly improve the structural strength of the material.

Therefore, the Young’s modulus of pristine Si/C is 17.2 GPa. After single-solid-phase coating (Si/C@Pitch), it increases to 20.4 GPa, indicating that the pitch-derived carbon skeleton alone contributes a modulus increment of 3.2 GPa (≈18.6% of the total increment in the solid–liquid system). After single-liquid-phase coating (Si/C@RGFQ), the Young’s modulus reaches 26.5 GPa, meaning the RGFQ-derived carbon network alone contributes a modulus increment of 9.3 GPa (≈54.1% of the total increment in the solid–liquid system). For the solid–liquid coated sample (Si/C@Pitch+RGFQ), the Young’s modulus further increases to 30.3 GPa, with a total increment of 13.1 GPa relative to pristine Si/C. The synergistic increment (beyond the sum of individual contributions) is 0.6 GPa, which arises from the interface strengthening between the rigid skeleton and flexible network. This confirms that the rigid pitch skeleton provides foundational strength, while the flexible RGFQ network dominates modulus enhancement, with synergistic effects further optimizing mechanical performance.

After being subjected to external pressure, the powder particles are not tightly packed under low-pressure conditions, resulting in high porosity between the powders. As the external force increases, the powder particles flow and rearrange to form a tightly packed state, and the porosity between particles also decreases accordingly. When the external load exceeds the yield strength of the material, some particles enter the plastic flow stage, resulting in irreversible and continuous reduction in the porosity between the powders; at the same time, the brittle particle system will undergo fragmentation, and the particle pore size will significantly decrease. The actual compression process of powder is a complex composite process, where elastic deformation coexists with plastic deformation. Elastic deformation can be restored, while plastic deformation is irreversible. The compression performance of powders is the focus of research on the mechanical properties of powders, but in the field of lithium-ion batteries, more attention is often paid to the compression performance of finished batteries. Therefore, this article will study the compression performance of powder materials as part of the characterization of mechanical properties. The schematic diagram of the testing equipment and compression performance testing function is shown in Figure 5e. Test parameters: Apply a pressure of 10–200 MPa to the material in sequence using the upper pressure head, with an interval of 20 MPa, and maintain the pressure for 10 s. The compression of material particles is accompanied by elastic deformation and plastic deformation. When the pressure applied to the powder particles is relieved, the particles will recover from elastic deformation. The thickness of the powder after relief minus the thickness after compression is defined as the rebound thickness of the powder. Figure 5f shows the pressure variation curve of the difference in rebound thickness between different materials. It can be seen from the figure that as the pressure increases, the rebound thickness of the material gradually increases and tends to stabilize. The rebound thickness of Si/C@Pitch+RGFQ is the largest, indicating that the material has large elastic deformation and can withstand high pressure. Figure 5g shows the compression of material thickness with increasing pressure. It is evident from the figure that the thickness of all materials gradually decreases with increasing pressure. Combined with the mechanism of the powder compression process, when the powder itself breaks, irreversible plastic deformation accounts for a large proportion, and the rebound thickness of the material cannot be restored after unloading. The characterization of powder particle breakage is achieved through the unloading test mode.

The performance of lithium-ion batteries is closely related to their internal resistance, which is mainly composed of ion resistance and electronic resistance. Electronic resistors mainly include negative electrode active material resistance, current collector resistance, contact resistance between active materials, contact resistance between active materials and current collectors, and electrode ear welding resistance. The precise quantitative analysis of the conductivity characteristics of electrode components and base materials has a decisive impact on improving the predictive efficiency of the overall impedance parameters of energy storage units [58]. Figure 5h shows the testing principle of the BER resistance meter, which uses upper- and lower-plane controllable pressure probes to directly measure the thickness direction of the pole piece and obtain the overall resistance of the pole piece. Figure 5i shows the conductivity characteristics of the polarizer. As the testing pressure increases, the conductivity of the polarizer shows a significant upward trend. This phenomenon can be attributed to the tighter contact between polar particles under high-voltage conditions, which shortens the conductivity path between particles, promotes effective charge transfer, and thus enhances conductivity. Specifically, the Si/C@Pitch+RGFQ sample exhibits the highest conductivity, indicating that the gaps between particles in the sample are relatively small, forming an excellent conductive network.

### 3.4. Half-Cell Performance

**Figure 6:**
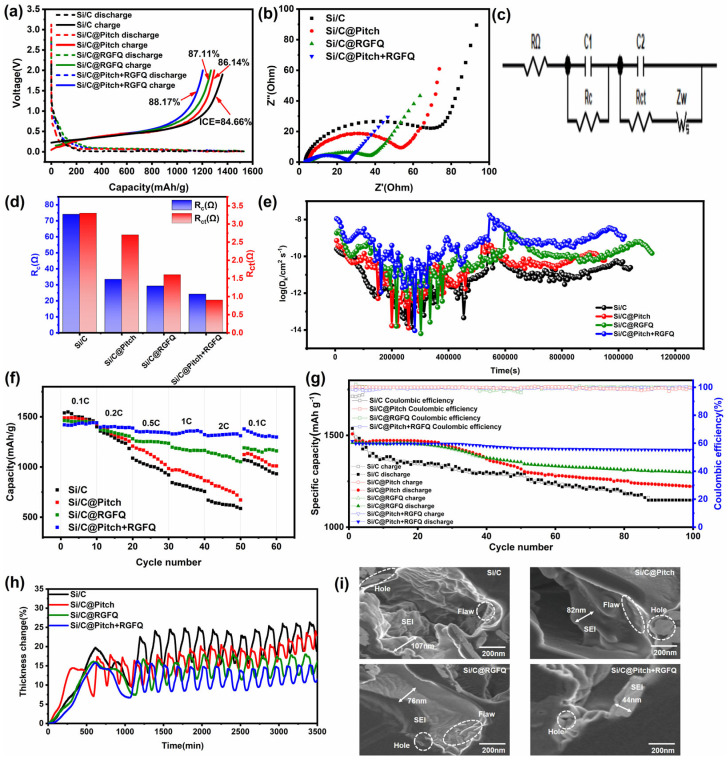
(**a**) Half-cell first charge and discharge curves of all samples. (**b**) Cyclic voltammetry curve of sample electrochemical impedance spectroscopy. (**c**) Equivalent circuit diagram of EIS. (**d**) Material contact impedance and interface impedance diagram. (**e**) Distribution diagram of diffusion coefficient. (**f**) Discharge performance at different rates. (**g**) 100 charge–discharge cycle curves. (**h**) Thickness variation curve. (**i**) SEM image of the polarizer after 100 cycles.

Initial charge and discharge tests were conducted on Si/C raw materials and Si/C samples coated with carbon sources. Figure 6a shows the required capacity voltage relationship curve at a test voltage range of 0.005–2 V and a current density of 0.1 C (1 C = 1.5 A g^−1^). From the graph, it can be seen that the reversible specific capacity and initial coulombic efficiency (ICE) of the Si/C raw material are 1580.6 mAh g^−1^ and 84.66%, respectively. At the same time, the reversible specific capacity of all coating materials (Si/C@Pitch, Si/C@RGFQ, and Si/C@Pitch+RGFQ) decreases to a certain extent due to the increase in carbon content and the decrease in silicon content. Meanwhile, the ICE of all coating materials has increased. From Figure 2a, it can be seen that the rough surface of the original Si/C particles causes the electrolyte to penetrate into the interior and form a large amount of SEI film inside and outside the particles, which consumes more Li^+^ and leads to a decrease in ICE. When surface defects are repaired by the carbon layer, ICE is significantly improved after a rapid decrease in specific surface area. Therefore, it can be inferred that the specific surface area of the material is one of the most important reasons for its improvement in ICE. The smaller the SSA, the smaller the formation of SEI film. In addition, the SEI film formed on the surface of the carbon layer is thinner than the SEI film on silicon, thereby reducing the consumption of Li ions and improving ICE. In addition, it is necessary to achieve the optimal balance between the two important goals of high specific capacity and high first efficiency. One should not be sacrificed for the other, especially if too much carbon is added in pursuit of high first efficiency, which not only sacrifices specific capacity but also increases preparation costs. Therefore, obtaining high-quality surfaces with small carbon increments is very important, and the solid–liquid coating method has significant advantages in this regard. The ICE of Si/C@Pitch+RGFQ is the highest (88.17%).

Electrochemical impedance spectroscopy (EIS) was performed on different Si/C samples, and the obtained figure is shown in Figure 6b. Figure 6c provides an equivalent circuit diagram. Electrochemical impedance spectroscopy is usually represented by the Nyquist plot. This characterization method decomposes the impedance of the system into a vector synthesis of the real part (resistance component) and the imaginary part (capacitance characteristic), where the *y*-axis represents the absolute value of the capacitive impedance. The test data shows that the impedance response curves of the four samples all exhibit dual characteristic regions: the high-frequency domain capacitance arc corresponds to the charge transfer process and the interface double-layer effect, and the low-frequency domain shows a 45 ° characteristic diagonal line reflecting the solid-phase diffusion control mechanism. The semicircle in the mid- to high-frequency range represents the impedance (R_SEI_) and interface impedance (R_ct_) of the SEI passivation film [3,59], and the diagonal line in the low-frequency region represents the Warburg impedance (Z_w_) caused by the diffusion of lithium ions inside the material, where the diameter of the semicircle in the high-frequency region reflects the transmission impedance of charges at the electrode interface. The larger the diameter of the arc, the greater the impedance and the more difficult the charge transmission [60,61]. After carbon coating on the surface of Si/C, the interface impedance significantly decreased, indicating that carbon coating effectively improved the electron transfer efficiency and thus increased the conductivity. From the figure, it can be seen that the interface impedance of Si/C raw material is relatively high, and that of Si/C@Pitch+RGFQ is the smallest, which is conducive to the diffusion of Li^+^ in the electrode material [62]. From Figure 6d, it can be seen that Si/C has the highest R_ct_ of 74.1 Ω and a contact impedance (R_c_) of 3.3 Ω, which is due to the influence of exposed silicon on electron transfer efficiency. With the completion of coating, surface defects are repaired, and the R_ct_ of Si/C@Pitch+RGFQ is the smallest, at 23.2 Ω, and its R_c_ is 0.7 Ω, indicating better charge transfer rate. From the figure, it can be seen that after covering the Si/C surface with a carbon layer, the interface impedance significantly decreases, indicating that the carbon coating effectively improves the electron transfer efficiency, thereby increasing the conductivity. Meanwhile, the interface impedance of the samples prepared by solid–liquid coating method is always the lowest. This indicates that the solid–liquid coating method produces the least number of defects, which is consistent with the previous conclusion that the specific surface area of the Si/C@Pitch and Si/C@RGFQ materials is always greater than that of Si/C@Pitch+RGFQ. This also confirms that Si/C@Pitch+RGFQ can better repair surface defects of Si/C composite materials than Si/C@Pitch and Si/C@RGFQ, resulting in a denser and less defective carbon layer.

The lithium-ion diffusion coefficients of the three materials were tested by the constant current intermittent titration method. Figure S1a–d show the GITT curves of Si/C, Si/C@Pitch, Si/C@RGFQ, and Si/C@Pitch+RGFQ. According to Formula (S1) in the Supplementary Materials, the time-dependent curve of the lithium-ion diffusion coefficient in Figure 6e was calculated. From the figure, it can be seen that the lithium-ion diffusion coefficients of Si/C@Pitch, Si/C@RGFQ, and Si/C@Pitch+RGFQ are generally higher than those of Si/C composite materials, indicating that the carbon layer on the surface of the material after coating can increase the lithium-ion diffusion coefficient of the material, thereby enhancing its rate performance.

The rate discharge performance of the material was tested at current densities of 0.1 C, 0.2 C, 0.5 C, 1 C, 2 C, and 0.1 C, and the results are shown in Figure 6f. From the graph, it can be seen that the average reversible capacity of Si/C at 0.1 C, 0.2 C, 0.5 C, 1 C, and 2 C is, respectively, 1474.2, 1288.6, 1001.2, 831.7 and 624.4 mAh·g^−1^, When the magnification was reset back to 0.1 C, the capacity recovered to 1171.8 mAh g^−1^, with a capacity loss of 25.8%. This is due to the electrode collapsing after cycling, the material not being coated, and a layer of SEI film forming on the surface of Si particles. After cycling at different current densities, a large number of cracks and a thick SEI film appeared on the surface of the Si negative electrode, and some electrodes on the material surface even peeled off, resulting in severe electrode cracking. Si/C@Pitch+RGFQ exhibits the best magnification performance. When the charging and discharging rate is reset back to 0.1 C, it exhibits a reversible capacity close to complete recovery, which is 1316.8 mAh·g^−1^, with a capacity loss of 7.5%. This indicates that the rate performance of the material can be greatly improved after coating, and Si/C@Pitch+RGFQ can better withstand the huge volume expansion of the Si negative electrode during lithium insertion, avoiding severe electrode breakage and reducing the loss of negative electrode active material. Therefore, during the rapid charging and discharging process, the electrode can better adapt to the volume expansion of Si material, thereby ensuring the capacity utilization of the material. This indicates that the electrode is minimally affected in rate performance testing.

Figure 6g shows the room-temperature cycling performance of all samples in a half cell with lithium sheets as the negative electrode. From the figure, it can be seen that after coating the carbon layer, the cycling performance significantly improved compared to the Si/C raw material (black line). The cyclic curve of Si/C@Pitch+RGFQ is smoother, indicating more stable performance during 100 cycles. In addition, materials prepared by the solid–liquid coating method have good cycling performance. This means that high-quality surfaces reduce the amount of SEI film produced, thereby reducing electrolyte decomposition, improving interface stability, maintaining the stability of the conductive environment, and contributing to the cycling performance of the material. After 100 cycles, the capacity retention rate of Si/C is only 74.6%, while the capacity retention rate of Si/C@Pitch+RGFQ is 92.4%. However, it can also be seen that the specific capacity of the material decreases with increasing carbon content. Due to the excessive electrolyte in half cells, the impact of electrolyte depletion is not significant, and further verification of cycling performance is needed through commercial cylindrical full cells.

One of the purposes of the particle-coated carbon layer is to utilize the mechanical properties of the carbon layer to constrain the excessive volume changes caused during the lithiation process. This not only damages the composite particles, electrodes, and reduces cycling performance, but also causes the battery shell to expand, posing a safety hazard. Therefore, the volume expansion of Si/C composite materials is an important indicator for their application. The prepared half-cell sample was installed in an in situ electrode thickness tester. First, a 0.2 C charging and discharging cycle was performed, followed by a 0.5 C charging and discharging process to systematically observe the dynamic changes in electrode thickness. Figure 6h clearly shows the test results: at a testing time of up to 3500 min, the uncoated Si/C electrode exhibited the maximum expansion rate of 26.3%. It is worth noting that in the 1271 min of testing, there was a sudden increase in the expansion rate due to the fracture phenomenon caused by the expansion of the pole piece. At 2890 min, the pole piece broke again and the expansion continued to intensify. After carbon coating treatment, the expansion rate of the Si/C@Pitch electrode was reduced to 23.2%. However, at 2890 min, the pole piece also broke and expanded continuously. In contrast, the Si/C@Pitch+RGFQ polarizer exhibited more stable performance, with expansion rates controlled at lower levels of 13.2%. What is particularly prominent is that Si/C@Pitch+RGFQ has the smallest thickness change after charge–discharge cycles, thanks to the excellent mechanical properties of its carbon layer. This carbon layer can effectively absorb the internal stress generated by the volume changes of silicon materials during charging and discharging, thereby significantly suppressing the expansion and contraction of silicon particles, enhancing the cycling stability of the electrode, and potentially extending the service life of the battery.

Figure 6i shows the surface SEM images of all sample electrodes after 100 cycles. It can be clearly seen from the figure that after 100 cycles, all electrodes are covered by a thick SEI film, and cracks appear on the Si/C electrode, resulting in the thickest SEI film (107 nm). This is because the surface of Si/C particles is rough, with a large number of cracks, pores, and exposed silicon (the peak intensity of Si 2p is the highest, reaching 8.7%), and the electrolyte easily penetrates into the interior and reacts violently with silicon. During the lithium insertion process, the volume expansion of silicon causes the SEI film to repeatedly rupture and rebuild, forming a thick and unstable SEI film (as shown in Figure 6i, there are obvious cracks and thick layers of SEI film on the surface of the uncoated electrode). Exposed silicon particles undergo anisotropic volume expansion (~200%) during lithiation, leading to particle fracture and electrode cracking (Figure 6h,i). This structural damage destroys the conductive network, increasing contact resistance (R_c_ = 3.3 Ω, Figure 6d). Rough surface defects and exposed silicon promote excessive electrolyte decomposition, forming a thick, brittle SEI film. Repeated expansion/contraction causes SEI rupture, triggering continuous Li^+^ consumption and capacity fade (capacity retention = 74.6% after 100 half-cell cycles, Figure 6g). After solid-phase, liquid-phase, or solid–liquid coating, the carbon layer covers surface defects, reducing direct contact between silicon and the electrolyte. The Si/C@Pitch+RGFQ surface has the lowest silicon content (significantly reduced Si 2p peak intensity), and the carbon layer is dense and has fewer defects (the I_D_/I_G_ value is the lowest, 0.87). The SEI film mainly forms on the surface of the carbon layer, with a thin and stable thickness (only a thin SEI film can be seen on the surface of the coated sample in Figure 6i, without obvious cracks). The main reason is that the dense carbon layer (an 11 nm thick amorphous carbon layer) blocks the penetration of electrolyte and reduces the contact area between silicon and the electrolyte. After coating, the specific surface area (SSA) significantly decreased (the lowest SSA was achieved by the solid–liquid coating method), the number of surface reaction sites decreased, and the amount of SEI film generated decreased. The high-stiffness carbon layer (Young’s modulus 30.3 GPa) constrains the volume expansion of silicon, reduces the rupture and reconstruction of SEI film caused by mechanical stress, and maintains the thin and stable state of the film. Therefore, Si/C@Pitch+RGFQ is the most capable of withstanding volume changes, adapting to the volume changes of silicon, and releasing mechanical stress.

Figure S2a–c compare the electrochemical performance (ICE, cycling performance, rate performance) of [63,64,65,66,67] with the materials prepared in this paper. In [63], a carbon source was used as a polymer for coating Si/C composite materials. In [64], the CVD method was used for coating, and the carbon source was C_3_H_6_. In [65], CVD and spray drying methods are used for coating, and the carbon source is polymer. In [66], the surface carbon layer was prepared by coating Si/C composite material with polyvinyl-butyral (PVB)-based carbon and then high-temperature carbonization. In [67], pitch is used as a carbon source to load a carbon layer on the surface of silicon, and porous silicon–carbon anode materials are prepared by etching the carbon layer and silicon with sodium hydroxide. Compared with Si/C materials coated by similar methods reported in the literature, this paper presents a more complete advantage in the dimensions of ICE, cycle performance, and rate performance, with better coordination of multiple indicators, forming a performance “envelope” covering the literature materials. This indicates that the material in this paper is more outstanding in terms of energy utilization efficiency, cycle stability, and charge–discharge rate adaptability, especially regarding Si/C@Pitch+RGFQ materials prepared using dual carbon sources.

### 3.5. Mechanism Analysis of Solid–Liquid Coating Superiority

The solid–liquid coating method (Si/C@Pitch+RGFQ) combines the unique advantages of solid-phase pitch (Pitch) and liquid-phase RGFQ (RGFQ) to form a composite carbon layer that combines rigidity and flexibility. Its superiority stems from the following collaborative mechanisms:

(1) Pitch forms a rigid carbon skeleton with a polyaromatic ring structure after high-temperature carbonization, and its intrinsic Young’s modulus is high (the graphitized carbon layer can reach~1000 GPa), providing high-strength support for the carbon layer. The carbon layer thickness of Si/C@Pitch in the article is 18 nm, but there are many defects (I_D_/I_G_ = 0.96). However, solid–liquid coating significantly reduces the defect rate (I_D_/I_G_ = 0.87) by introducing liquid-phase resin to fill its pores, maximizing the mechanical confinement ability of the rigid skeleton.

(2) RGFQ is dried by a spray in liquid form and evenly coated with Si/C particles. Its small molecular chains can penetrate into nanometer-scale pores and form an amorphous carbon network after carbonization. This “capillary infiltration effect” increases the contact area between the carbon layer and the surface of Si particles, enhances the interfacial bonding strength (XPS shows that the intensity of the Si 2p peak decreases to its lowest), and thus suppresses the volume expansion of silicon (the expansion rate decreases from 26.3% to 13.2%).

(3) Raman spectroscopy shows that the I_D_/I_G_ value (0.87) of solid–liquid coating is lower than that of single-solid-phase (0.96) or liquid-phase (0.93) coating, indicating that the sp^2^ hybridized carbon in the carbon layer has a higher degree of order and fewer defects (such as vacancies and dislocations). This is because the high-temperature carbonization of pitch promotes graphitization, while resin carbonization fills lattice defects, forming a dual effect of “repair strengthening”. The XRD results showed that the diffraction peak width of Si was the smallest after solid–liquid coating, indicating that the recrystallization of amorphous Si was suppressed at high temperatures (average grain size of 12.8 nm), which was attributed to the “geometric constraint” effect of the dense carbon layer, limiting the growth of silicon grains.

(4) The rigid pitch skeleton bears the main compressive stress, while the flexible resin carbon network absorbs the shear stress generated by the volume expansion of silicon. The AFM force–displacement curve shows that the elastic deformation of Si/C@Pitch+RGFQ (6.7 nm) is much smaller than that of a single coating, indicating that it can buffer volume changes through the “elastic energy storage release” mechanism and avoid electrode cracking.

The superiority of the solid–liquid coating method is essentially the result of the multi-scale synergy of “carbon source synergy structure optimization interface regulation”. Through the design of a composite carbon layer that combines rigidity and flexibility, it synchronously solves the core problems of volume expansion, interface instability, and poor conductivity of silicon-based negative electrodes, providing a theoretical basis and process paradigm for the preparation of high-performance Si/C composite materials.

### 3.6. Full-Cell Performance

Half-cell testing is a conventional method for evaluating the electrochemical properties of anode and cathode materials, especially when using lithium metal sheets as the negative electrode. It is particularly critical for analyzing parameters such as the first coulombic efficiency, impedance characteristics, and cycling curve of the material. Although half-cell testing can also be used to evaluate the cycling stability of materials, the presence of a large amount of liquid electrolyte in the system often masks the problem of decreased negative electrode cycling performance caused by rapid electrolyte consumption. In view of this, a commercial 18650 cylindrical lithium-ion battery was used as the testing platform to comprehensively evaluate key indicators such as cycling performance, high- and low-temperature discharge performance, and rate performance of the materials in the entire battery. Due to the fact that the specific capacity of all samples exceeds 1200 mAh·g^−1^, their large volume expansion makes direct application of current lower capacity commercial cathodes complex. Therefore, these materials are typically combined with commercial graphite (CG) to achieve a target specific capacity of 420 mAh·g^−1^, adjusting the mixing ratio: Si/C:CG = 6.1:93.9, Si/C@Pitch:CG = 6.6:93.4, Si/C@RGFQ:CG = 6.8:93.2, Si/C@Pitch+RGFQ: CG = 7.1: 92.9.

**Figure 7:**
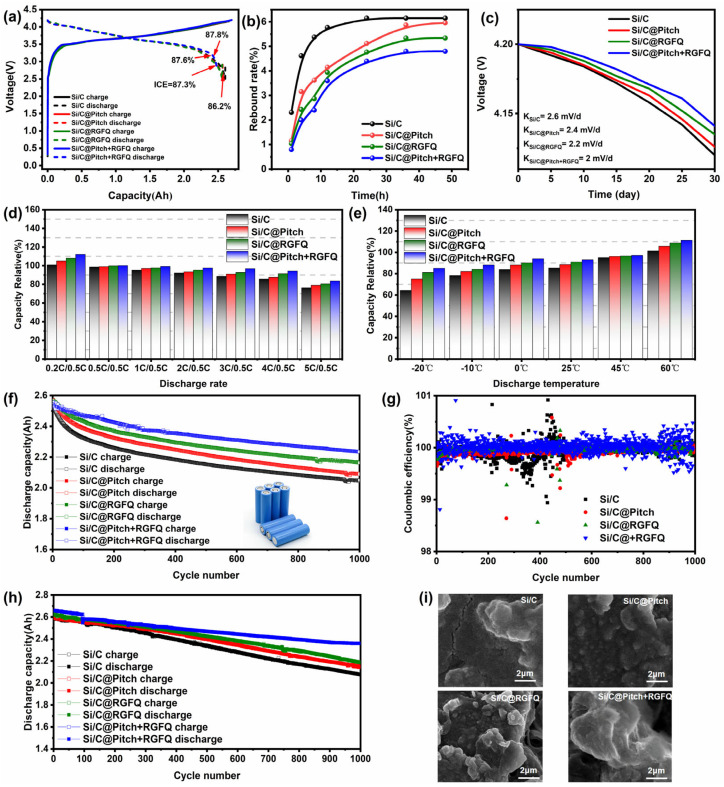
(**a**) First charge–discharge curves of the full-cell, (**b**) polar rebound rate, (**c**) polar rebound rate, (**d**) discharge performance at different rates, (**e**) discharge performance at different temperatures, (**f**) 1000 charge–discharge cycles, room temperature (25 °C) discharge performance, (**g**) coulombic efficiency at room temperature, (**h**) 1000 charge–discharge cycles, high temperature (45 °C) discharge performance, and (**i**) SEM image of electrode surface after 1000 cycles of room temperature charge and discharge.

The full-cell test evaluated the battery within the voltage range of 2.5–4.2 V, with a charging current density of 0.5 C and a discharging current density of 0.5 C. The initial charge–discharge curves of all cylindrical battery samples in Figure 7a show that the charging capacity of the battery prepared this time remained around 2.6 Ah. Due to the combination of the prepared material and commercial graphite, the initial coulombic efficiency of the latter half of the battery was between 86 and 87%, lower than that of NCM811. Therefore, the initial coulombic efficiency of this test was displayed as the negative electrode material, and the consistency was good. Figure 7b shows the changes in rebound rate of each polarizer within a 48 h time range. From the graph, it can be observed that during the time period of 1–24 h, the Si/C electrode exhibits the maximum rebound rate, with a value of 10.2%. Subsequently, after 24 h, the rebound rate of the polarizer gradually stabilized without any further increase, and its final maximum rebound rate was determined to be 10.89%. In contrast, Si/C@Pitch, Si/C@RGFQ, and Si/C@Pitch+RGFQ showed the greatest rebound within the 1–12 h time period. It is worth noting that after 32 h, the rebound phenomenon of the Si/C@Pitch+RGFQ polarizer basically stops, and the final rebound rate is the smallest among the three, only 7.9%. This result not only demonstrates the advantage of the Si/C@Pitch+RGFQ polarizer in terms of structural stability, but also further validates the effectiveness of its preparation process. To evaluate the performance stability of the battery under static conditions, the battery was left to stand at room temperature for 30 days and its voltage was measured every 5 days. By observing the changes in battery voltage over time, one can indirectly determine the battery’s wear and performance. Figure 7c shows the relationship between time and voltage. From the data in the figure, it can be clearly seen that the voltage of all batteries in the initial state is 4.2 V. However, during the 30-day static process, the voltage drop rate of the Si/C battery is the most significant, and after 30 days, its voltage drops to 4.117 V. In contrast, the battery treated with carbon coating has a smaller voltage drop during the static period; the Si/C@Pitch+RGFQ battery maintains a voltage of 4.131 V after 30 days, showing good voltage stability. The self-discharge K value is a key indicator for measuring the self-discharge rate of lithium-ion batteries, which reflects the voltage drop of the battery within a specific time interval. The battery made of Si/C has the highest K value (2.6 mV/d), indicating a faster self-discharge rate. In contrast, the Si/C@Pitch+RGFQ battery has the smallest K value (2 mV/d), indicating a lower self-discharge rate and superior performance. The results indicate that the carbon layer confinement structure effectively buffers the volume deformation of high-capacity silicon-based active materials through the elastic deformation mechanism. Its three-dimensional network architecture exerts isotropic mechanical confinement on silicon grains, controlling the volume expansion rate within a reasonable range and significantly improving the self-discharge performance of the material.

We performed rate performance testing on the battery, set the working potential range to 2.5–4.2 V, used constant current charging mode (0.5 C), and then performed constant current discharge testing with gradually increasing current densities (0.2 C, 0.5 C, 1 C, 2 C, 3 C, 4 C, and 5 C). By comparing the ratio of the released electricity under different current conditions to the reference value (0.5 C), the capacity retention data was collected, as is shown in Figure 7d. When using the reference current density (0.5 C), the system exhibits the optimal capacity retention rate (98.7%). This is attributed to the lower current carrying intensity, which prolongs the carrier migration time window and promotes the full release of internal charges in the active material. When the current density increases to 5 C, the capacity retention rate decreases, which is closely related to the relaxation time of carrier transport. During the rapid deintercalation process, some ions fail to reach the current collector interface in a timely manner due to diffusion rate limitations, resulting in a decrease in the utilization rate of effective active substances. It is worth noting that the Si/C@Pitch+RGFQ sample shows a relatively small decrease in capacity, and even with an increase in discharge rate from 0.5 C to 5 C, its relative capacity change remains at a low level. Specifically, at a high discharge rate of 5 C, the relative capacity of Si/C@Pitch+RGFQ samples is the only one among all tested samples that exceeds 80%. This result indicates that the carbon layer on the surface of the sample effectively acts as an electron channel, improving the charge transfer efficiency in the material [68]; therefore, the Si/C@Pitch+RGFQ full cell exhibits excellent rate performance, maintaining high capacity stability even under fast discharge conditions, without a rapid decrease due to an increase in discharge rate. We performed discharge tests on the battery at different temperatures (−20 °C, −10 °C, 0 °C, 25 °C, 45 °C, 60 °C) using standard charge–discharge mode (cut-off voltage 2.5–4.2 V, charge–discharge rate of 0.5 C) for constant current cycling testing. From Figure 7e, it can be seen that as the temperature increases, the capacity performance of the entire battery gradually improves. At a low temperature of −20 °C, the capacity of the Si/C sample can only be utilized at 63%, while in contrast, the Si/C@Pitch+RGFQ sample achieves 86% capacity utilization. The temperature dependence of electrochemical performance is due to the synergistic effect of multi-scale transport mechanisms. Under sub normal temperature conditions (−20 °C to 0 °C), the rheological properties of the electrolytic system deteriorate, with its viscosity increasing by 3.2 times compared to normal temperature, resulting in a decrease in the lithium-ion migration number. This mass transfer barrier directly leads to a 67% increase in charge transfer impedance, significantly limiting the effective capacity of the battery. This material can maintain good capacity performance over a wide temperature range, demonstrating excellent high-temperature and low-temperature adaptability, and showing good commercial prospects. Its capacity stability over a wide temperature range can meet the needs of most application scenarios. We performed a cyclic test on the battery with a voltage range of 2.5–4.2 V, a charging current density of 0.5 C, and a discharging current density of 0.5 C. From Figure 7f, it can be seen that the Si/C material has the worst full-cell cycling performance, with only 2 Ah of charging capacity remaining after 1000 cycles and a capacity retention rate of only 80.4%. After coating the carbon layer, the cycling performance is improved and the capacity retention rate is significantly increased. It is worth noting that Si/C@Pitch+RGFQ has the best cycling performance, with a capacity retention rate of up to 82.3% after 1000 cycles. This is because Si/C@Pitch+RGFQ has the lowest SSA and the highest ICE; however, an increase in its carbon content also leads to a significant decrease in specific capacity. The coulombic efficiency of the full cell after 1000 cycles at room temperature in Figure 7g also indicates that the coated sample with better surface quality has better cycling stability than the Si/C material. This phenomenon indicates that the surface carbon layer not only improves the ICE of Si/C materials, but also significantly enhances their cycling performance. The carbon layer can effectively cover the exposed Si particles on the surface of Si/C material and block the pores, cracks, and other defects leading to the interior, thereby hindering direct contact between the electrolyte and the surface and interior Si particles, reducing the generation of SEI film, and slowing down the consumption rate of the electrolyte [69]. At the same time, the surface carbon layer provides a buffer layer for the volume change of Si/C material, reducing the impact of particle breakage and thus improving the cycling performance to a certain extent. Under the same high-temperature condition of 45 °C for charge–discharge cycling, the data in Figure 7h shows that under high-temperature conditions, the initial discharge specific capacity reaches 2.65 Ah, which is 6% higher than for the 25 °C room-temperature system. This activation effect originates from the reduction in dissociation energy in the solvation layer of lithium ions, which promotes charge exchange kinetics. However, during long-term cycling, the high-temperature system exhibits significant capacity degradation. After 1000 cycles, the capacity of the Si/C battery decays to 1.8 Ah, with a capacity retention rate of about 76.7%, which is attributed to the structural degradation of the electrode material under high temperature. Although Si/C@Pitch+RGFQ has a lower capacity retention rate than at room temperature, it still exhibits good cycling performance, with a capacity retention rate of up to 81.3%, indicating that Si/C@Pitch+RGFQ maintains stable full-cell cycling characteristics under high specific capacity conditions. Figure 7i shows the polar scanning electron microscopy images of Si/C, Si/C@Pitch, Si/C@RGFQ, and Si/C@Pitch+RGFQ samples after 1000 cycles. Through comparative analysis, we can draw the following conclusion: for the Si/C sample, the figure clearly shows that cracks and pores appeared on its surface after 1000 cycles. These structural damages directly demonstrate that the material has a lower lifespan after long-term charge–discharge cycles, making it prone to structural damage and affecting the performance and stability of the battery. In contrast, due to the carbon layer coating treatment applied to the Si/C@Pitch, Si/C@RGFQ, and Si/C@Pitch+RGFQ materials, almost no cracks or pores are visible on their surfaces. Instead, only the formation of SEI film on the surface can be observed. In Figure 7i, cracks appeared on the uncoated Si/C electrode after cycling, while the carbon-coated electrode had no cracks. The main reasons for this are as follows: the uncoated Si/C surface is rough and has many defects, forming a “point contact” structure after compaction, with high internal porosity. The volume expansion of silicon during cycling (~200%) causes pore collapse, and mechanical stress concentration leads to cracks. Uncoated Si reacts directly with the electrolyte to form a thick and unstable SEI film (107 nm), which repeatedly ruptures/rebuilds with changes in silicon volume and accelerates electrode structure collapse in conjunction with expansion stress (thickness expansion rate of 26.3%). The solid–liquid coated carbon layer (Young’s modulus 30.3 GPa) controls the silicon expansion rate at 13.2% through a “rigid flexible composite” structure, uniformly disperses stress, and suppresses crack formation. The dense carbon layer reduces surface silicon content (<5%, Figure 4d) and oxygen content (8.1%, Figure 4i), minimizing electrolyte penetration and SEI formation. The thin, stable SEI film reduces Li^+^ consumption, contributing to high ICE and long-term cycle stability (82.3% retention after 1000 full-cell cycles, Figure 7f). Uniform preparation process parameters were used to eliminate the influence of process differences, confirming that the cracks were caused by the synergistic effect of volume expansion and SEI film rupture during cycling. In summary, carbon coatings suppress electrode cracks through mechanical constraints, interface stability, and structural homogenization, and multidimensional data confirms their advantages. This observation strongly demonstrates the importance of carbon coating in improving the cycling performance of negative electrode materials.

## 4. Conclusions

This study constructed a carbon layer on the surface of Si/C composite materials using three coating processes and systematically compared the effects of different methods on the surface quality, mechanical properties, and electrochemical properties of the materials. The main conclusions are as follows:

1. Surface quality and structural optimization: The solid–liquid coating method (Si/C@Pitch+RGFQ) significantly improved the surface defects of Si/C materials, forming a uniform and dense amorphous carbon layer (with a thickness of 11 nm). Compared with the solid-phase coating method and liquid-phase coating method, the coating material obtained the lowest specific surface area (SSA) and carbon increment. The surface silicon content (<5%) and oxygen content (8.1%) were significantly reduced, effectively suppressing electrolyte penetration and excessive SEI film formation.

2. Improvement in mechanical properties: Compared with solid-phase coating and liquid-phase coating methods, the carbon layer of solid–liquid coating has the highest Young’s modulus (30.3 GPa), which combines high stiffness and elastic deformation ability. It can effectively constrain the volume expansion of silicon-based materials during charging and discharging (the expansion rate drops to 13.2%), reduce particle breakage and electrode structure damage, and improve the structural stability of the material.

3. Electrochemical performance advantages: This material exhibits excellent electrochemical performance, with a first coulombic efficiency (88.17%) and cycle stability (92.4% capacity retention rate for 100 cycles) that are significantly better than for the single-coating method. The entire battery exhibits good capacity retention ability over a wide temperature range (−20 °C to 60 °C) and high rate (5 C), with capacity retention rates of 82.3% (room temperature) and 81.3% (45 °C) after 1000 cycles.

4. Method value and application prospects: The solid–liquid coating method combines the synergistic effect of solid-phase asphalt and liquid-phase RGFQ to achieve efficient and uniform coating of the carbon layer, improving overall performance while reducing costs. From the perspective of technological scalability, this method has significant advantages: Firstly, at the raw material level, both pitch and RGFQ are low-cost carbon sources for industrial mass production, with stable supply and controllable procurement costs. Secondly, the potential for scalability is significant. Compared to high-end coating technologies such as CVD that rely on vacuum equipment, the solid–liquid coating method has lower equipment investment and energy consumption. It is easier to meet the cost and efficiency requirements of industrial mass production. The research results not only provide a key technical approach for surface modification of Si/C composite materials but also accelerate the commercialization process of high-energy density and long-cycle-life lithium-ion batteries due to their good scalability. They have important application value in fields such as energy storage power stations, electric vehicles, and aerospace, where battery performance and cost are strictly regulated.

## Supplementary Materials

The following supporting information can be downloaded at https://www.mdpi.com/article/10.3390/nano15171300/s1, Figure S1: GITT curve for (a) Si/C, (b) Si/C@Pitch, (c) Si/C@RGFQ, (d) Si/C@Pitch+RGFQ; Figure S2: Radar comparison of electrochemical performance between Si/C carbon coated materials and references [57,58,59,60,61]: (a) Si/C@Pitch, (b) Si/C@RGFQ, (c) Si/C@Pitch+RGFQ.
