# Supplementary material for: Preparation of High-Strength and High-Rigidity Carbon Layer on Si/C Material Surface Using Solid–Liquid Coating Method
Source: Nanomaterials (Basel). 2025 Aug 22;15(17):1300. doi: 10.3390/nano15171300 (PMC12430276; doi:10.3390/nano15171300)
Supplement: Supplementary file 1 [file nanomaterials-15-01300-s001.zip › nanomaterials-3804279-supplementary.pdf]

nanomaterials

Supplementary Material for

Preparation of High-Strength and High-Rigidity Carbon Layer on Si/C Material Surface Using Solid-Liquid Coating Method

This file includes Fig. S1 to S2.

Galvanostatic Intermittent Titration Technique (GITT) is an experimental method designed based on Fick's second law, aimed at quantitatively evaluating the diffusion rate of ions in electrode materials. This method is based on a core assumption that the diffusion process is mainly limited to the surface region of the material. The GITT technology mainly consists of two key stages: firstly, applying a short-term constant current pulse for discharge. To ensure that the diffusion process is limited to the surface layer, the duration t of the pulse needs to be strictly controlled to meet the condition t<<L2/D_k. The entire GITT testing process consists of a series of cycles of "pulse application constant current discharge static relaxation", through which the diffusion characteristics of ions in electrode materials can be accurately measured and calculated. This article uses the Xinwei testing system for testing. The diffusion coefficient D_k of Li^+ during the first charge discharge lithiation process of the material at room temperature is expressed as:

$$D_k = \frac{4m_b V_m}{\pi M_b S \Delta t}\left(\frac{\Delta E_s}{\Delta E_\tau}\right)^2 \quad (1)$$

In the formula, m_b represents the amount of active material in the silicon oxygen electrode, M_b represents the molar mass of the silicon oxygen material, V_m represents the molar volume of the active material, S represents the geometric area of the silicon oxygen negative electrode, Δt represents the lithiation time, ΔE_s is the total voltage change caused by the pulse, and ΔE_τ is the voltage change during constant current charging/discharging.

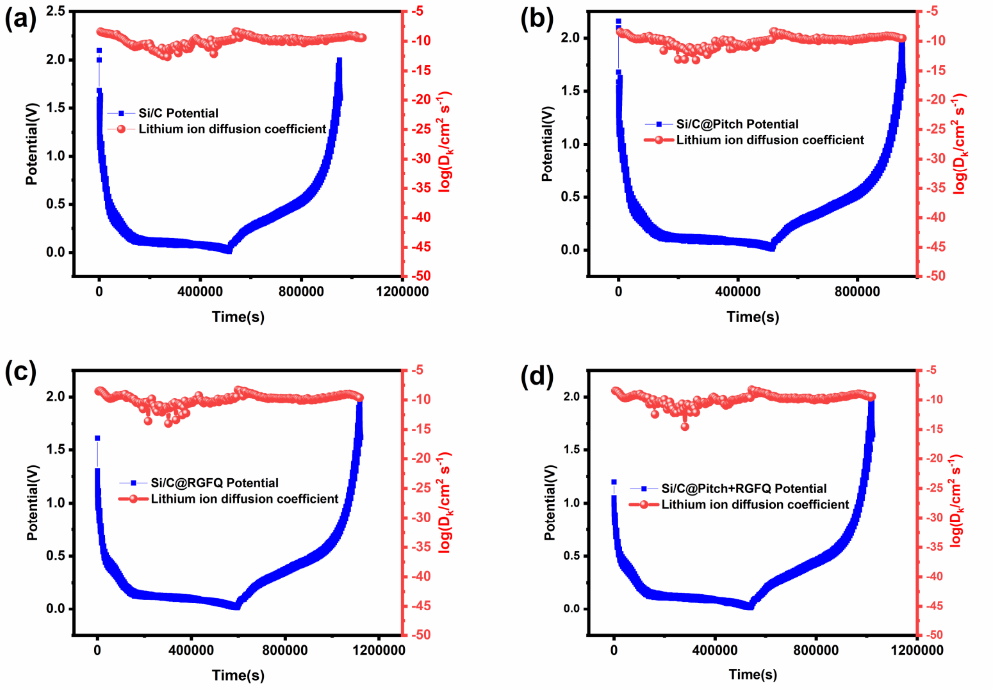

Figure S1. GITT curve for (a) Si/C, (b) Si/C@Pitch, (c) Si/C@RGFQ, (d) Si/C@Pitch+RGFQ.

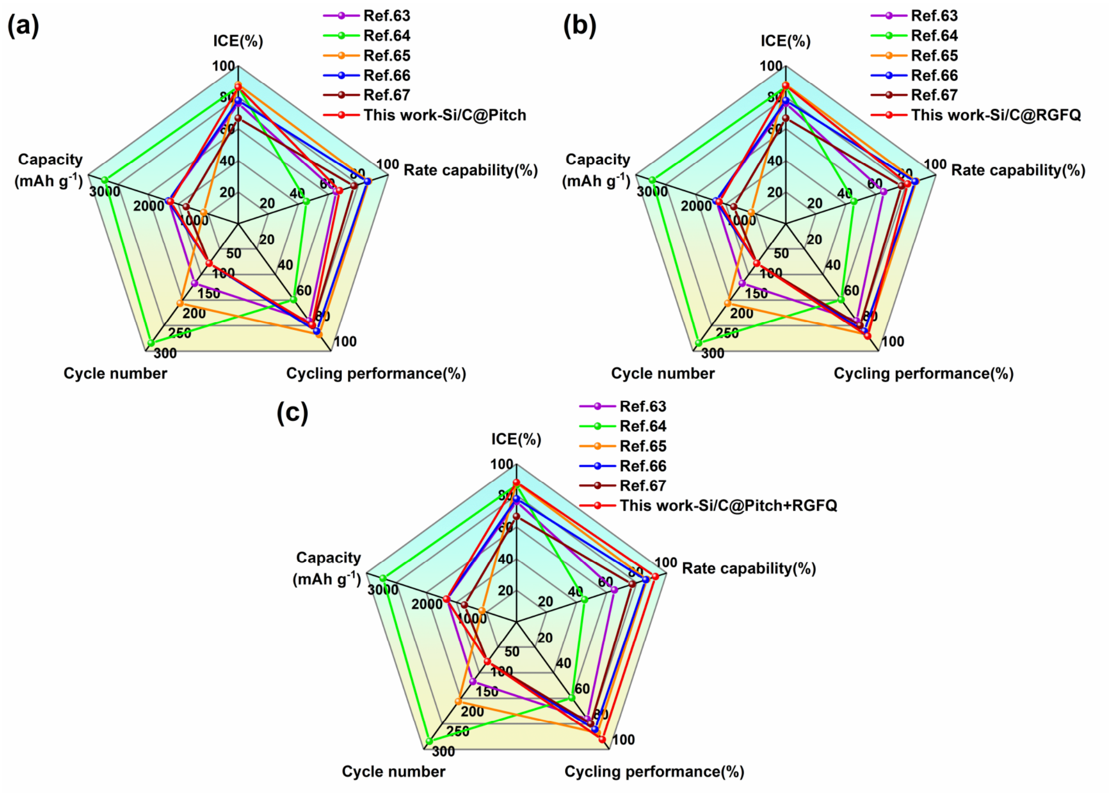

Figure S2. Radar comparison of electrochemical performance between Si/C carbon coated materials and references 57-61: (a) Si/C@Pitch, (b) Si/C@RGFQ, (c) Si/C@Pitch+RGFQ.
